# Pediatric Melanoma and Drug Development

**DOI:** 10.3390/children5030043

**Published:** 2018-03-20

**Authors:** Klaus Rose, Jane M. Grant-Kels

**Affiliations:** 1 klausrose Consulting, Pediatric Drug Development & More, 4125 Riehen, Switzerland; 2 Department of Dermatology, University of Connecticut Health Center, Farmington, CT 06030, USA; grant@uchc.edu

**Keywords:** pediatric drug development, Pediatric Investigation Plan (PIP), pediatric pharmaceutical legislation, EU pediatric regulation, pediatric clinical pharmacology, developmental pharmacology, pediatric laws, pediatric clinical studies

## Abstract

Importance—Pediatric melanoma occurs, albeit rarely. Should patients be treated by today’s medical standards, or be subjected to medically unnecessary clinical studies? Observations—We identified international, industry-sponsored pediatric melanoma studies triggered by regulatory demands in www.clinicaltrials.gov and further pediatric melanoma studies demanded by European Union pediatric investigation plans. We retrieved related regulatory documents from the internet. We analyzed these studies for rationale and medical beneficence on the basis of physiology, pediatric clinical pharmacology and rationale. Regulatory authorities define children by chronological age, not physiologically. Newborns’ organs are immature but they develop and mature rapidly. Separate proof of efficacy in underage patients is justified formally/regulatorily but lacks medical sense. Children—especially post-puberty—and adults vis-a-vis medications are physiologically very similar. Two adolescent melanoma studies were terminated in 2016 because of waning recruitment, while five studies in pediatric melanoma and other solid tumors, triggered by European Union pediatric investigation plans, continue recruiting worldwide. Conclusions and Relevance—Regulatory-demanded pediatric melanoma studies are medically superfluous. Melanoma patients of all ages should be treated with effective combination treatment. Babies need special attention. Children need dose-finding and pharmacokinetic studies but adolescents metabolize and respond to drugs similarly to adults. Institutional Review Boards/ethics committees should suspend ongoing questionable pediatric melanoma studies and reject newly submitted questionable studies.

## 1. Introduction

Metastatic malignant melanoma, once incurable, can today be treated, even in some cases with durable responses [1]. Pediatric melanoma has three major challenges: (1) differentiation between conventional adult-type and other melanoma types [2]; (2) differentiation between melanoma simulants that are seen more often in children than in adults (spitz nevi) [2]; and (3) treating patients appropriately despite their age [3]. The Food and Drug Administration (FDA) and the European Medicines Agency (EMA) promote and require pediatric studies [4,5,6]. In melanoma, such studies appear to recruit predominantly patients with adult-type conventional melanoma [7]. A new definition of underage patients in the context of pharmaceutical treatment should be considered, together with a thorough revision of drug approval in the various pediatric age groups.

## 2. Methods

We identified terminated and ongoing international, industry-sponsored pediatric melanoma studies in www.clinicaltrials.gov, excluding studies that recruit(ed) both adolescents and adults to focus on truly pediatric studies. However, we included studies that recruited children, adolescents and young adults up to 30 years of age. We retrieved related EMA/FDA documents from the internet. We identified further pediatric melanoma studies demanded by the EMA in pediatric investigation plans (PIPs). We analyzed the regulatory rationale and medical beneficence of terminated, ongoing and demanded studies on the basis of physiology, pediatric clinical pharmacology and reason. PIP decisions and www.clinicaltrials.gov-listed studies can be internet-retrieved by their respective number.

### Background

Pediatric melanoma is rare but is captured in registries [8,9,10] and is eagerly discussed [2,3,11,12,13,14,15,16,17]. The literature describes it in patients from <10 to ≤21 years of age [3]. Should adolescents be therapeutically considered children? The American Academy of Pediatrics (AAP) defines children in the context of healthcare as ≤21 years but accepts pediatric healthcare for older patients with special needs [18]. Although these age ranges are appropriate for hands-on pediatric clinical care, they should not be used to limit access to pharmacological treatment.

The claim that children are discriminated against evolved after US law, in 1962, established clinical trials as the basis for regulatory drug approval, a principle now recognized worldwide [19]. Also, jurisdiction over prescription drug advertising was transferred to the FDA [20]. In the 1950s, drug toxicities in newborns had been reported [21,22]. From 1962 onwards, drug developers included pediatric warnings on drug labels to avoid potential lawsuits. As a result, these drugs could not be advertised for children. Shirkey claimed this denied children the use of drugs and characterized children as “therapeutic orphans” [23]. The AAP claimed that drug prescription for children without explicit FDA certification was experimental [24] and that children needed separate pharmacological evaluation of new drugs for all age groups [25]. The AAP guideline of 1995 [25] explicitly referenced the toxicities reported in the 1950s [21,22]. FDA and AAP lobbying resulted in a 1997 law that rewarded pediatric studies with voluntary “pediatric exclusivity” of an additional six months’ protection against generic competition [4]. When a company submits a proposal and the FDA agrees, it issues a “Written Request” (WR). After study report submission and FDA scrutiny, pediatric exclusivity is granted [4]. A second law authorized the FDA to mandate pediatric studies without reward [4]. Both laws are now permanent [26].

This inspired the European Union (EU) to establish its own pediatric regulation, in effect since 2007 [4,27]. Without a pediatric investigation plan (PIP), new drugs cannot get adult EU-approval, unless the targeted disease is PIP-exempted [4,5,6]. PIPs must address juvenile animal studies, formulations (e.g., tablets vs. syrup), pediatric studies and more. The EMA has until now issued >1000 PIPs [28].

The reported toxicities had been in premature newborns [21,22]. Also, further toxicities reported later under the sensational sub-headline “CONTINUED PEDIATRIC THERAPEUTIC DISASTERS” listed until the 1980s only toxicities in preterm newborns and newborns [29]. The AAP warnings were, in our opinion, inappropriately extended to all children by “extrapolating” toxicities from physiologically immature newborns to all children. Pediatric laws responded to the AAP’s “moral imperative to formally study drugs in children so that they can enjoy equal access to existing as well as new therapeutic agents” [25]. Children were defined not based upon physiology but administratively: FDA < 16 [30], EU < 18 years [4,6,27].

## 3. Results

In 2008, the EMA withdrew adolescent melanoma from the list of PIP-exempted diseases [5]. Thirteen melanoma PIPs were issued (Table 1). Twelve PIPs demand systemic monotherapy studies in melanoma or solid tumors including melanoma [5], this includes the original ipilimumab melanoma PIP [31]. The talimogene PIP demands local injection into melanoma and other non-central nervous system (CNS) malignant solid tumors. Two PIP decisions that originally demanded pediatric studies [32,33] were later changed into waivers (no pediatric studies demanded).

Melanoma PIPs require variably PK data in patients aged from 6 months to 17 years, 1–17, 12–17, ≤24 and ≤30 years; for ipilimumab, nivolumab, paclitaxel, pembrolizumab and trametinib they demand randomized comparisons [5]. Talimogene is an oncolytic for direct injection into unresectable melanoma tissue [34]. The talimogene PIP demands two studies on injection into melanoma tissue or other advanced non-CNS tumors in “pediatric” patients aged 2–17 years.

In 2008, the US National Cancer Institute (NCI) initiated the first pediatric ipilimumab study [35]. In 2011, the FDA approved ipilimumab for melanoma [36] and the EMA issued an ipilimumab melanoma PIP [31]. In 2014, the FDA issued an ipilimumab WR (see Table 2) [37]. The developer negotiated with both the EMA and the FDA and provided ipilimumab for the first pediatric NCI study [35]. EMA and FDA incorporated the NCI study into their pediatric demands (EMA)/written request (FDA). The NCI study was completed with 33 patients—including 12 melanoma patients—and was published [38]. The PIP/WR ipilimumab clinical study #2 was initiated, listed on www.clinicaltrials.gov [39] and eventually reported [40].

Two industry-sponsored international melanoma studies in adolescents were terminated in 2016 because recruitment had waned, predominantly due to now available combination treatment (Table 3) [41]. The ipilimumab study had been both WR/PIP-requested [5], the vemurafenib study PIP-demanded (Table 3) [42]. After the vemurafenib study was terminated, the EMA changed the vemurafenib PIP decision (Table 3) into a waiver, although the original decision had already triggered a clinical study [42].

Five industry-sponsored, PIP-demanded studies in children, adolescents and young adults with melanoma and other tumors are ongoing (Table 4). For studies’ centers, see Table 5.

## 4. Discussion

Industry-sponsored pediatric melanoma studies are driven by the FDA/EMA in the spirit of the “therapeutic orphans” concept. These studies are regulatorily justified on the assumption of two distinct populations, adult versus pediatric, each requiring separate studies. Both US and EU laws claim concern for child health but closer inspection of key documents shows a regulatory rationale. For the FDA, a good example is the shift of wording from the FDA’s first pediatric report to congress in 2001 to the second one in 2016. In 2001, it described expected clinical outcomes: “quicker recoveries from childhood illnesses, with fewer attendant hospital stays, physician visits and parental work days lost” [43]. In 2016, it reported “significant progress in terms of the number, timeliness and successful completion of studies of drugs in pediatric populations” [44]. This a shift away from clinical concerns towards a regulatory justification of pediatric studies. Also, the EMA wording suggests clinical concern, for example, their brochure entitled “Better Medicines for Children” [45]. However, the focus in this brochure is the authorization of drugs, not clinical care. The EMA 10-year report on EU pediatric regulation [46] in comparison to its recent publication by EMA employees [47] demonstrates their true motivation.

We find it questionable that the EMA required pediatric clinical studies in the first vemurafenib melanoma PIP [33] but thereafter changed the PIP decision into a full waiver (no pediatric studies required, Table 3), without a public explanation for why it had first required the developing company to initiate a study [42] and then waived this decision. Additionally, there are several clinical studies listed in www.clinicaltrials.gov for talimogene laherparepvec for various cancer types. If one of them should show statistically significant superiority, Amgen would ask for approval for a new indication. However, there is no medical merit in requiring Amgen to recruit 18 young patients to participate in an international clinical study with 17 centers (see Table 4 and Table 5) for various non-central nervous system tumor types, including melanoma. All these patients have in common is that they are young (Table 4). These 18 patients are being studied because of a regulatory authority enforcing pediatric studies. Meanwhile these patients are not being advantaged of the newer combination therapies that are available.

Children and adolescents with adult-type conventional melanomas [2] should be treated with proven medications that are dose-adjusted for their weight and physiology. In preterm newborns, newborns and babies, absorption, distribution and excretion (ADME) are quite different. After roughly the first year of life, ADME becomes comparable to adults [48]. Children need PK- and dose-finding studies, not separate proof of efficacy. Adolescents have mature bodies as far as pharmaceutical treatment is concerned. Patients of all ages deserve treatment with effective anti-melanoma combinations. This holds true even for the very rare cases of melanoma in babies and newborns, provided conventional melanoma is sufficiently differentiated from other melanoma types [2]. Institutional Review Boards (IRBs)/ethics committees (ECs) should not have approved the terminated studies (Table 3). The “pediatric” phase 1 ipilimumab study in children, adolescents and young adults [35,38] continued even after ipilimumab approval. After ipilimumab approval in 2011 this was no longer experimental, no longer a phase 1 study and had not been a pediatric study from the beginning.

Within pediatric academic clinical oncology, an international industry has evolved that is dedicated to FDA/EMA-promoted “pediatric” studies, as reflected in the number of study centers and planned patients in Table 3, Table 4 and Table 5. Not all clinical researchers involved in international pediatric melanoma studies are aware of the regulatory background. Participation in international studies offers prestige, networking, investigators’ meetings, opportunities to publish and more. Certainly, the flow of funds is welcome. These funds are channeled by regulatory decisions. The regulatory authorities have been given much credit in promoting pediatric research but the studies we discuss here are to a large degree not pediatric studies and are without clinical beneficence. The conflicts of interest of regulatory authorities and pediatric researchers have so far been barely addressed in the scientific literature. It is time for medicine and academia to address this blind spot.

## 5. Conclusions

IRBs/ECs should immediately suspend, worldwide, questionable monotherapy melanoma studies and reject newly submitted unnecessary pediatric studies. Melanoma patients from suspended studies should be offered combination treatment; other patients should be offered appropriate therapy, not monotherapy based on questionable regulatory requirements for pediatric studies. IRBs/ECs should uphold the Belmont report’s principle of beneficence [49].

## Figures and Tables

**Table 1:** European Medicines Agency (EMA) melanoma Pediatric Investigation Plans (PIPs).

Compound	PIP Number
Binimetinib	EMEA-001454-PIP03-15
Cobimetinib	EMEA-001425-PIP01-13-M01
Dabrafenib	EMEA–001147-PIP01-11-M03
Encorafenib	EMEA-001588-PIP01-13
Ipilimumab *	EMEA-000117-PIP02-10 [31] • EMEA-000117-PIP02-10-M07
MAGE-A3 recombinant protein **	EMEA-001099-PIP02-11 [32] • EMEA-001099-PIP02-11-M01
Nivolumab	EMEA-001407-PIP01-12
Paclitaxel	EMEA-001308-PIP01-12
Pembrolizumab	EMEA-001474-PIP01-13
Selumetinib	EMEA-001585-PIP01-13
Talimogene laherparapvec	EMEA-001251-PIP01-11-M03
Trametinib	EMEA-001177-PIP01-11-M02
Vemurafenib **	EMEA-000978-PIP01-10 [33] • EMEA-000978-PIP01-10-M01

* The first ipilimumab melanoma PIP, EMEA-000117-PIP02-10 is retrievable through the EMA document library [31]. Its current version M07 (7th modification) can be retrieved through Google. ** PIPs later changed into waivers (no pediatric studies required). Original PIPs can be retrieved by the EMA document library, the respective link is referenced; current PIP versions can be googled by its respective number.

**Table 2:** First two ipilimumab Written Requested (WR) clinical studies.

An open label, dose-escalation study of ipilimumab in pediatric patients (aged 1–21 years) with refractory cancers. A clinical study of ipilimumab in pediatric patients (12–<18 years) with unresectable or metastatic melanoma to evaluate PK and safety. Efficacy in adolescent patients (12–<18 years) will be determined by extrapolation from results observed in adult patients treated with ipilimumab for unresectable or metastatic melanoma.

**Table 3:** Terminated industry-sponsored international studies in adolescents with melanoma.

Study #	Abbreviated Study Description	Centers	Sponsor	Pts	Age (y)	PIP/WR
NCT01519323	Vemurafenib in Stage IIIC/IV Melanoma with BRAFV600 Mutations	26	Roche	6	12–17	EMEA-000978-PIP01-10 [34]
						EMEA-000978-PIP01-10-M01
NCT01696045	Ipilimumab in untreated or previously treated advanced or metastatic melanoma.	32	BMS	12	12–17	EMEA-000117-PIP02-10 (Original) [31]
						EMEA-000117-PIP02-10-M07 (current) WR [37]

Abbreviations: Pts—patients; Roche—Hoffman-La Roche; BMS—Bristol-Myers Squibb; y—years.

**Table 4:** Ongoing industry-sponsored pediatric studies including patients with melanoma.

Study #	Abbreviated Study Description	Centers	Age	Pts	Sponsor	PIP #
NCT02332668	Pembrulizumab in advanced melanoma or advanced R/R PDL1–positive solid tumors or lymphoma	45	6-month–17 y	310	MSD	EMEA-001474-PIP01-13
NCT01677741	Dabrafenib in advanced BRAF V600 mutation–positive solid tumors	27	1–17 y	86	GSK	EMEA–001147-PIP01-11-M03
NCT01962103	Paclitaxel DF & PE in R/R solid tumors	20	6-month–17 y Ph1	107	Celgene	EMEA-001308-PIP01-12
			2–24 y Ph2			
EUdraCT 2014-004685-25	Cobimetinib DE, S & PK in previously treated solid tumors	41	1–17 y (DES)	50	Roche	EMEA-001425-PIP01-13-M01
			6–30 y (ES)			
NCT02756845	S&E of talimogene laherparepvec in melanoma and advanced non-CNS tumors	17	12–21 y Ph1	18	Amgen	EMEA-001251-PIP01-11-M03
			2–11 y Ph2			

Abbreviations in alphabetic order: CNS—central nervous system; DE—dose escalation; DES—dose escalation study; DF—dose finding; ES—expansion study; GSK—GlaxoSmithKline; MSD—Merck, Sharp & Dome; PE—preliminary; Ph1—phase 1; Ph2—phase 2; PK—pharmacokinetics efficacy; Pts—Patients; Roche—Hoffman-La Roche; R/R—recurrent or refractory; S—safety; y—years.

**Table 5:** Study centers of terminated and ongoing “pediatric” melanoma studies.

Study #	Compound	Study Centers
NCT01519323	**Vemurafenib**	US: Los Angeles (CA), Aurora (CO), St. Peterburgh (FL), Bethesda (MD), Boston (MA), New York (NY), Memphis (TN), Houston (TX) • Australia: Westmead, Brisbane • France: Marseille, Pierre Benite • Germany: Kiel, Mainz, Tuebingen • Israel: Jerusalem, Petach-Tikva • Italy: Roma, Genova, Milano • Poland: Wroclaw • Slovakia: Bratislava • Spain: Esplugues De Llobregat-Barceona, Sevilla • UK: Newcastle, Sutton
NCT01696045	**Ipilimumab**	US: Phoenix (AZ), Los Angeles (CA), Orange (CA), Aurora (CO), Tampa (FL), Indianapolis (IN), Boston (MA), Rochester (MN), New York (NY), Pittsburg (PA), Memphis (TN), Houston (TX), 2 × Salt Lake City (UT) • Belgium: Gent • Denmark: Copenhagen • France: Lyon, Marseille, Nantes, Villejuif Cedex • Germany: Dortmund, Erlangen, Hamburg, 2 × Muenster • Mexico: 2 × Mexico DF, Leon • Spain: Esplugues de Llobregat-Barcelona • UK: Bristol, Newcastle, Sutton
NCT02332668	**Pembrulizumab**	US: Phoenix (AZ), Loma Linda (CA), 2 × Los Angeles (CA), Madera (CA), Orange (CA), San Diego (CA), San Francisco (CA), Aurora (CO), New Haven (CT), Washington DC, Atlanta (GA), Indianapolis (IN), Iowa City (IA), Boston (MA), Ann Arbor (MI), 2 × Minneapolis (MN), Cansas City (MO), Sant Louis (MO), New York (NY), Cincinatti (OH), Cleveland (OH), Columbus (OH), Philadelphia (PA), Pittsburg (PA), Memphis (TN), Nashville (TN), Dallas (TX), Fort Worth (TX), Houston (TX), Salt Lake City (UT), Seattle (WA), Milwaukee (WI) • Australia: North Ride • Brazil: Sao Paulo • Canada: Kirkland • France: Paris • Germany: Haar • Israel: Hod Hasharon • Italy: Rome • Korea: Seoul • New Zealnd: Wellinton • Sweden: Stockholm • UK: Hoddesdon
NCT01677741	**Dabrafenib**	US: Phoenix (AZ), Orange (CA), Baltimore (MD), Boston (MA), New York (NY), Cincinatti (OH), Memphis (TN), Seattle (WA) • Australia: Parville, Subiaco • Canada: Toronto • Denmark: Copenhagen • France: Marseille, Paris cedex 05, Paris cedex 12, Toulouse, Villejuif cedex • Germany: Heidelberg, Regensburg, Berlin • Israel: Jerusalem, Ramat-Gan • Italy: Milano • Spain: Esplugues de Llobregat-Barcelona, Madrid • UK: Sutton, London
NCT01962103	**Paclitaxel**	US: Phoenix (AZ), New York (NY) • Canada: Ontario • France: Lyon, Nancy, Paris, Villejuif • Italy: Firenze, Genova, Milano, Padvoa, Rome, Torino • Spain: 2 × Barcelona, Madrid, Sevilla, Valencia • Switzerland: Zuerich • UK: Sutton
EUdraCT 2014-004685-25 *	**Cobimetinib**	Netherlands • Ireland • Denmark • UK • Germany • Spain
NCT02756845	**Talimogene laherparepvec**	US: Wilmington (DE), Chicago (IL), Indianapolis (IN), Detroit (MI), NY (NY), Cincinatti (OH), Columbus (OH) • Canada: Montreal • France: Lyon, Marseille, Paris • Spain: Barcelona, Esplugues de Llobregat, Valenica, Madrid • Switzerland: Basel, Zuerich

Explanations: Abbreviations for US states by two-letter codes of the US Postal Service. * www.clinicaltrialsregister.eu (EUdraCT) only lists countries, not individual study centers.
